# Microsatellites for Next-Generation Ecologists: A Post-Sequencing Bioinformatics Pipeline

**DOI:** 10.1371/journal.pone.0055990

**Published:** 2013-02-12

**Authors:** Iria Fernandez-Silva, Jonathan Whitney, Benjamin Wainwright, Kimberly R. Andrews, Heather Ylitalo-Ward, Brian W. Bowen, Robert J. Toonen, Erica Goetze, Stephen A. Karl

**Affiliations:** 1 Hawai‘i Institute of Marine Biology, University of Hawai‘i, Kāne‘ohe, Hawai‘i, United States of America; 2 Department of Oceanography, School of Ocean and Earth Sciences and Technology, University of Hawai‘i, Honolulu, Hawai‘i, United States of America; Emory University, United States of America

## Abstract

Microsatellites are the markers of choice for a variety of population genetic studies. The recent advent of next-generation pyrosequencing has drastically accelerated microsatellite locus discovery by providing a greater amount of DNA sequencing reads at lower costs compared to other techniques. However, laboratory testing of PCR primers targeting potential microsatellite markers remains time consuming and costly. Here we show how to reduce this workload by screening microsatellite loci via bioinformatic analyses prior to primer design. Our method emphasizes the importance of sequence quality, and we avoid loci associated with repetitive elements by screening with repetitive sequence databases available for a growing number of taxa. Testing with the Yellowstripe Goatfish *Mulloidichthys flavolineatus* and the marine planktonic copepod *Pleuromamma xiphias* we show higher success rate of primers selected by our pipeline in comparison to previous *in silico* microsatellite detection methodologies. Following the same pipeline, we discover and select microsatellite loci in nine additional species including fishes, sea stars, copepods and octopuses.

## Introduction

Microsatellite loci remain one of the most popular choices for population genetic studies. This success may be credited to several attributes including their ability to provide contemporary estimates of migration, distinguish relatively high rates of migration from panmixia, and resolving pedigrees [1]–[5]. In spite of their potential to address a myriad of issues in molecular ecology, evolution, and conservation, until recently the expertise, time and costs of initially developing microsatellite markers remained deterrents for many. This was particularly true for taxa where microsatellites tend to be relatively rare, such as in some insects, birds, bats and corals [6], [7]. Unfortunately, success rate of microsatellite marker development typically correlates with their frequency in the genome [8].

The technology of microsatellite development has recently undergone a revolution with massively parallel sequencing technologies (also known as next-generation sequencing or NGS) allowing large numbers of DNA sequences to be mined for microsatellite repeats. This approach has been successfully implemented to discover microsatellite loci in a growing number of species, including several previously recalcitrant taxa [9], [10], and even extinct taxa [11]. The longer read lengths of the 454 pyrosequencing platform (Roche 454 Life Science, Bradford CT, USA) have made it the preferred approach for microsatellites to date (but see [12]), and the continued rapid advances of NGS technology will make microsatellites even cheaper and easier to develop in the future.

The approaches of NGS microsatellite projects published to date have consisted of generating large amounts of sequencing data from microsatellite enriched libraries or genomic DNA [13], [14], which are then mined for microsatellite loci (typically thousands). Primers are designed from the region flanking the microsatellite and then are tested to identify markers with consistent PCR amplification of unique polymorphic loci [13]. Although this approach has greatly streamlined the microsatellite isolation process in comparison to previous lab methodologies that required cloning [15], [16], ample room remains for reducing the laboratory time and costs associated with post-sequencing marker development.

Can we increase the primer-to-marker conversion rate through selection of microsatellite loci via bioinformatics analysis? Focusing on three reef fishes, two sea stars, two copepods and two octopuses as case studies, we explore bioinformatic analyses to reduce the likelihood of the two most common pitfalls researchers encounter in the development of microsatellite markers: failed PCR amplification and unspecific amplification of multiple loci. Our study builds upon previous work [17], [18] to provide a pipeline to select microsatellite loci via post-sequencing bioinformatics analyses that emphasizes the importance of sequence quality, and avoids loci associated with repetitive elements. To evaluate our post-sequencing microsatellite selection (PSMS) bioinformatics pipeline, we compare the efficiency of our method to previous *in silico* microsatellite detection methodologies [17] demonstrating that we can streamline the development of microsatellite markers from next-generation sequencing.

## Materials and Methods

### Ethics Statement

Sample collection methods were approved by the Institutional Animal Care and Use Committee of the University of Hawai‘i (IACUC permit # 09-753-3 to B.W.B. and 10-816-3 to S.A.K).

### Preparation of Microsatellite Libraries

To obtain microsatellite markers by 454 pyrosequencing we followed two preparation methods: shotgun and microsatellite enrichment. In the shotgun method, genomic DNA was extracted from a fish fin clip (*Mulloidichthys vanicolensis* and *M. flavolineatus*), octopus muscle tissue (*Octopus cyanea* and *O. oliveri*) or whole copepods (*Haloptilus longicornis* and *Pleuromamma xiphias*) by one of two methods: (1) Qiagen DNeasy kits or (2) incubation with proteinase K at 56°C for 1 h followed by two extractions with phenol:chloroform:isoamyl alcohol (25∶24∶1), one extraction with chloroform:isoamyl alcohol (24∶1) and one ethanol precipitation. Using 500 ng of genomic DNA resuspended in water, we prepared the libraries and ligated different Multiplex Identifier (MID) adaptors to each library using the Rapid Library Preparation Kit following the manufacturer’s manual (454 Life Sciences). In the microsatellite enrichment method, genomic DNA was extracted from fish fin clips (*Paracirrhites arcatus*) or sea star tube feet (*Protoreaster nodosus* and *Acanthaster planci*) following method (2) described above. Microsatellite enriched libraries were made following [15], using *Rsa* I as the restriction enzyme. Four mixes of biotinylated oligonucleotides were used with mixture specific melting temperatures (*T*_m_) to enrich for microsatellites [Mix 1 (*T*_m_ = 50°C): (AAGC)_5_, (AACC)_5_, (AACG)_5_, (ATCC)_5_, (AAGG)_5;_ Mix 2 (*T*_m_ = 45°C): (ATC)_8_, (AAT)_10_, (AAC)_8_, G(AGG)_6_, (AAG)_8_, (ACG)_6_, G(CCG)_5_, (ACT)_8_, (ACC)_6_, (AGC)_6_; Mix 3 (*T*_m_ = 58°C): (TG)_10_, (TC)_10;_ and Mix 4 (*T*_m_ = 65°C): (AC)_12_, AG_12_]). Equimolar concentrations of microsatellite-enriched DNA obtained from each mix were pooled and purified using a QIAquick PCR cleanup kit (Qiagen, Valencia, CA, USA). Cleaned and enriched fragments were ligated with 454 sequencing primers and tagged with unique MID adaptors.

### Sequencing

Individually tagged library preparations were pooled in two mixes (see Table 1 for pooling summary) and each run on one fourth of a PicoTiterPlate and sequenced with titanium chemistry on a Roche GS-FLX sequencer (454 Life Sciences) at the Center for Advanced Studies in Genomics, Proteomics and Bioinformatics (University of Hawai‘i at Mānoa).

**Table 1:** Summary of the microsatellite libraries prepared in this study and sequencing results showing species name, library preparation method, pooling ratios indicated as portion of a PicoTiterPlate, sequence of the Multiplex Identifier (MID) adaptors and absolute and relative (%) numbers of reads obtained from each library.

Species	Method	Portion	MID	# Reads	Relative # Reads
**Sequencing Run 1**					
*Mulloidichthys vanicolensis*	shotgun	1/32	ACGCGTCTAGT	33962	11.3
*Mulloidichthys flavolineatus*	shotgun	1/32	ACGAGTAGACT	28135	9.4
*Paracirrhites arcatus* 1	enriched	1/32	ACACGTAGTAT	35945	8.6
*Paracirrhites arcatus* 2	enriched	1/32	ACACGACGACT	36551	12.2
*Protoreaster nodosus*	enriched	1/16	ACGACACGTAT	85404	28.4
*Acanthaster planci*	enriched	1/16	ACACTACTCGT	83835	27.9
Unmatched				6731	2.2
			Total	310563	
**Sequencing Run 2**					
*Haloptilus longicornis*	shotgun	1/16	ACGAGTAGACT	64916	24.7
*Pleuromamma xiphias*	shotgun	1/16	ACGCGTCTAGT	75702	28.7
*Octopus cyanea*	shotgun	1/16	ACGTACTGTGT	56233	21.4
*Octopus oliveri*	shotgun	1/16	ACGTACACACT	59445	22.6
Unmatched				7041	2.7
			Total	263337	

1 Pink morphotype,

2 Brown morphotype.

### Library Splitting and Adaptor and Primer Removal

We used the program Sff_extract (http://bioinf.comav.upv.es/sff_extract/) to extract the reads from the 454 SFF files and convert them into FASTQ files. We then split the individually tagged libraries and removed the 454 adaptors, MID tags and linkers using scripts from the Fastx_toolkit (http://hannonlab.cshl.edu/fastx_toolkit/).

### Post-sequencing Microsatellite Selection (PSMS)

For each data set, our goal was to increase the primer-to-marker conversion rate (i.e. the proportion of primer pairs that successfully amplify the desired product in PCR reactions). Our strategy is based on targeting sequence fragments with high base-call accuracy to be used as template for designing primers and avoiding targeting microsatellite loci associated with repetitive elements in the genome. In our quality control (QC) step, we used the software Clean_reads 0.2.1 (http://bioinf.comav.upv.es/clean_reads/index.html) to trim poor quality regions of the sequences following three steps. First, we removed low-quality bases from the ends of the sequence, second, we found regions of the sequence where the probability of error is highest. If regions with high error were found, the third step was to trim each of these sequences to the largest region having an average probability of error no greater than the maximum average error allowed (cut-off values listed below). The largest region meeting all of the criteria was chosen as the final clean read.

We applied two combinations of QC parameters in Clean_reads: i) a high stringency and ii) a low stringency. For (i) we removed any regions at either end or within a sequence read that within a 10 bp window had an average probability of error greater than 0.003, and trimmed each sequence read to the largest region having an average probability of error no greater than 0.003 (Clean_reads parameters: lucy_bracket = 10.0, 0.003; lucy_window = 10.0, 0.003; lucy_error = 0.003, 0.02). For (ii) we removed any regions at either end or within a sequence read that within a 10 bp window had an average probability of error greater than 0.02, and trimmed each sequence read to the largest region having an average probability of error no greater than 0.02 (Clean_reads default values: lucy_bracket = 10.0, 0.02; lucy_window = 50.0, 0.08; lucy_error = 0.025, 0.02).

Next, we performed a similarity analyses on the clean datasets to eliminate redundant sequences. Highly similar sequences were used to build contigs and reconstruct consensus sequences, with the help of the pipe1.pl and pipe2.pl scripts implemented in QDD2.1_beta (http://gsite.univ-provence.fr/gsite/Local/egee/dir/meglecz/QDD.html). Briefly, microsatellite containing sequences with perfect repeats of di- to hexanucleotides were selected, sequence similarity was detected by an all-against-all BLAST (BLAST-2.2.25+ ftp://ftp.ncbi.nih.gov/blast/executables/) and pairwise identity was calculated along the whole microsatellite flanking regions. According to this analysis the sequences were sorted as follows: sequences with >95% identity were grouped into contigs and consensus sequences were constructed with ClustalW-2.1.1 [19] (ftp://ftp.ebi.ac.uk/pub/software/clustalw2), where a minimum of 66% of the sequences had to have the same base at a site to accept it as a consensus. Sequences that fell under the 95% similarity threshold were eliminated from the pipeline, because of the increased risk of amplifying multiple loci or unspecific products. Sequences that had only hits to themselves were classified as singletons. Finally, we prepared a file containing all the singletons and a file with all the contigs.

We then identified sequences that showed similarity to known repetitive elements. To check for repetitive elements (RE), we compared both our singletons and contigs files against the RE database Repbase (Repbase v16.09; http://www.girinst.org/repbase/index.html). For the fishes we searched against repetitive elements in vertebrate genomes including transposable elements, pseudogenes, and integrated viruses (Repbase libraries were fugapp.ref, fugrep.ref, humrep.ref, humsub.ref, mamrep.ref, mamsub.ref, mousub.ref, prirep.ref, prisub.ref, pseudo.ref, ratsub.ref, rodrep.ref, rodsub.ref, synrep.ref, tmpxen.ref, vrtrep.ref, zebapp.ref, and zebrep.ref). For the sea stars, copepods and octopuses we searched against repetitive elements discovered in any animal genomes (same Repbase libraries as above plus the invertebrate libraries angrep.ref, drorep.ref, invrep.ref, cbrrep.ref, invsub.ref, celrep.ref, cinrep.ref, and cinunc.ref). Scanning was performed with Censor v4.2.27 [20] (http://www.girinst.org/downloads/software/censor/ ) using default sensitivity parameters. Sequences with >65% homology to known repetitive elements were excluded from further analysis. With the help of the script pipe3.pl of QDD2.1_beta we detected perfect repeats with a minimum length of five di- to hexanucleotide repeats. Using PRIMER3 v1.1.4 [21] (http://primer3.sourceforge.net/), primers were designed with microsatellite repeats as target regions to produce PCR products from 90 to 300 bp, and the minimum, optimum, and maximum oligonucleotide sizes set as 18, 20, and 27; minimum, optimum, and maximum *T*_m_ set at 57.0°C, 60.0°C, and 63.0°C; maximum difference in *T*_m_ for the primers of 1.0°C; minimum, optimum, and maximum GC content set at 20%, 50%, and 80%; and no GC clamp. Since 454 sequencing has high error rate at homopolymer sites, we set the maximum poly-X length to 4. All other parameters were set to the default values.

To evaluate the ability of our pipeline to increase primer-to-marker conversion rate, we synthesized 24 primer pairs for the goatfish *Mulloidichthys flavolineatus* and 15 primer pairs for the copepod *Pleuromamma xiphias* selected by our PSMS pipeline, and 20 and 15 primer pairs, respectively, from the QDD2.1_beta output. (The QDD pipeline includes neither the QC step nor the filter to eliminate sequences similar to repetitive elements deposited in Repbase). The PCR reactions were conducted using the M13-tailed primer method modified from [22] and described in [23], [24]. We optimized the PCR conditions as described in [25]. PCR amplification products were resolved using an ABI 3130 Genetic Analyzer and sized using GENEMAPPER v4.0 (Applied Biosystems).

## Results

### Pyrosequencing and Microsatellite Mining

In the first sequencing run, the six libraries generated 310563 DNA sequence reads with an average length of ∼550 bp. After barcode splitting we recovered 33962 and 28135 sequences for the goatfishes *M. vanicolensis* and *M. flavolineatus* (shotgun libraries), 25945 and 36551 for the libraries of arc-eye hawkfish *P. arcatus* (enriched libraries) and 85404 and 83825 for the sea stars *A. planci* and *P. nodosus* (enriched libraries; Table 1). Only 2.2% of the sequence reads remained unassigned, which was the result of sequencing errors in the barcodes themselves.

In the second sequencing run, the four libraries generated 263337 DNA sequence reads with an average length ∼550 bp. After barcode splitting we recovered 64916 and 75702 sequences for the copepods *H. longicornis* and *P. xiphias* (shotgun libraries) and 56233 and 59445 sequence reads for the octopus *O. cyanea* and *O. oliveri* (shotgun libraries). Only 2.7% of the reads remained unassigned. We mined the datasets for microsatellite repeats and in all cases we identified ∼6000, or more, loci in each of shotgun datasets and from ∼17000 to ∼23000 from enriched libraries (Table 1).

### Post-sequencing Selection of Microsatellite Loci Via Bioinformatics Analyses and Comparison to Previous Methodologies

**Figure 1:**
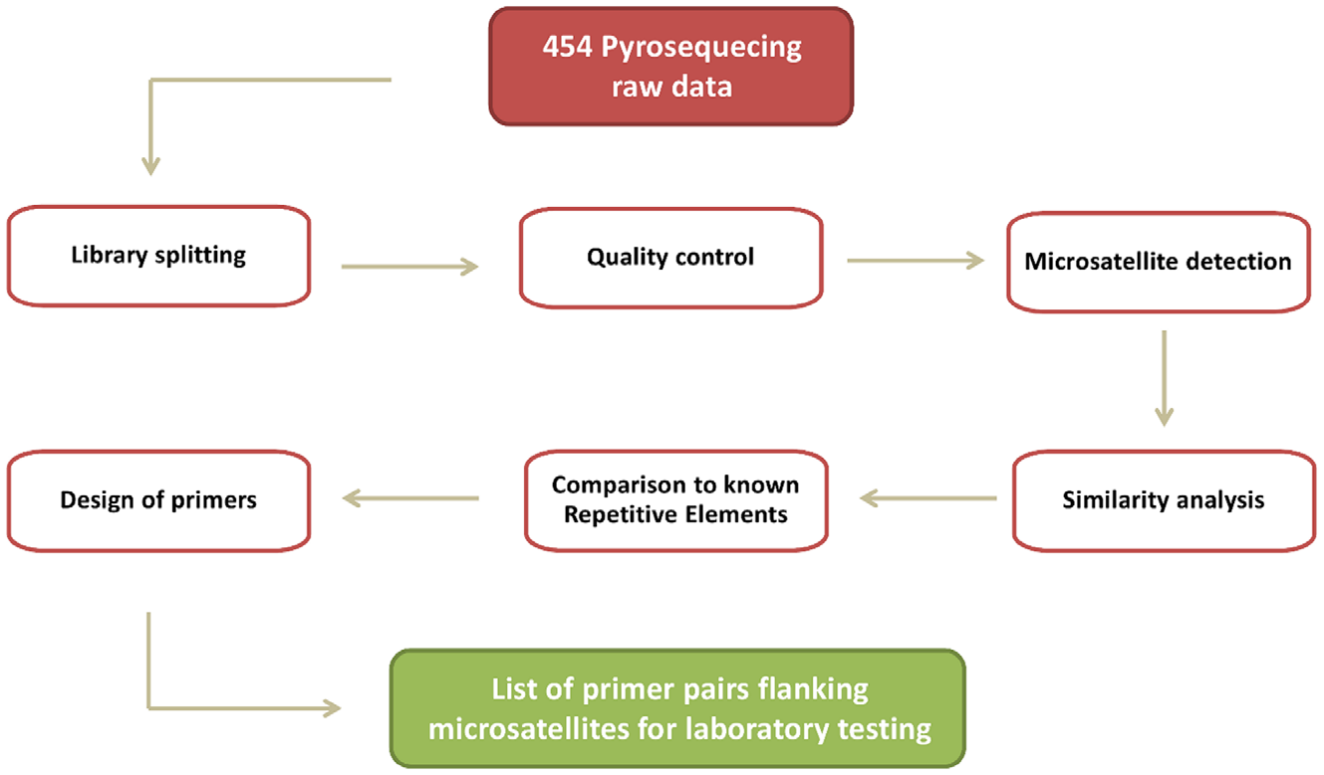
Post-sequencing microsatellite selection pipeline (PSMS).

Focusing on the reef fish *M. flavolineatus* as a case study, we generated a set of microsatellite primers for laboratory testing using previous *in silico* microsatellite detection methodologies ([17] implemented in QDD2.1_beta) versus our PSMS bioinformatic pipeline (Figure 1). Using QDD2.1_beta we searched 28135 *M. flavolineatus* DNA sequences and identified 6209 microsatellite loci with flanking sequences that allowed designing primer pairs with 90–300 bp target amplification products. In spite of thorough PCR optimization efforts, only two out of the 20 primer pairs that we designed and tested consistently yielded amplification products of the expected size.

For the PSMS pipeline we analyzed the 11960 highest-quality reads from *M. flavolineatus* (Table 2, Figure S1) and identified 1173 reads that had di- to hexanucleotide repeats. Of these, 937 reads had hits to only themselves (singletons) and were used in the remainder of the pipeline (see below). Additionally, 126 sequences were highly similar (>95% identity) to at least one other read and were grouped into 43 contigs. We also searched 27287 DNA sequence reads selected with less stringent QC filters and identified 5489 with microsatellite repeats, of which 752 were pooled into 256 contigs and were used in the remainder of the pipeline. Additionally, we identified 4291 sequences as singletons.

**Table 2:** Summary numbers for the post-sequencing selection of microsatellite loci from sequencing run 1.

Number	*M. flavolineatus*	*M. vanicolensis*	*P. arcatus* 1	*P. arcatus* 2	*P. nodosus*	*A. planci*
**Before Quality Control**						
Sequences	28135	33962	35945	36551	85404	83835
µsats	8302	5953	22844	19468	18278	17734
% with µsats	29.5	17.5	63.6	53.3	21.4	21.2
**After Quality Control**						
Low Stringency - consensus						
Sequences	27287	33186	33507	33551	81474	77352
µsats	256	189	983	695	677	368
µsats without RE^3^	225	163	817	593	558	318
Primer pairs	176	135	526	442	374	231
High Stringency - singletons						
Sequences	11960	16459	8062	8773	24093	12403
µsats	937	732	1467	1279	328	132
µsats without RE^3^	701	559	1048	938	264	103
Primer pairs	286	245	349	408	92	45
Total primer pairs	462	380	875	850	466	276

1 Pink morphotype.

2 Brown morphotype,

3 RE – repetitive elements.

For the rest of the pipeline we focused on the contig sequences (less stringent QC) and the singleton sequences (high stringency QC). For the contigs, we excluded 12% with similarity to vertebrate repetitive elements, leaving 225 contigs, 176 of which had flanking sequences that met the criteria for primer design (Table 2). For the singletons, we excluded 26.2% with similarity to repetitive elements, leaving 701 sequences, of which 286 were appropriate for microsatellite-flanking primer design. We synthesized 24 microsatellite primer pairs and tested them for reliable amplification. Twenty-three primer pairs selected by the PSMS pipeline showed consistent amplification of a unique product of the expected size, resulting in a 96% primer-to-marker conversion rate, compared to 10% with previous screening technology. We followed the same pipeline to discover and select 387 candidate microsatellite markers in the copepod *Pleuromamma xiphias* (Table 3). We synthesized and tested 15 of these primer pairs and in eight cases obtained a unique PCR amplification product of expected size, resulting in a 53% primer-to-marker conversion rate. These results contrast with the amplification success obtained when testing an equal number of primers synthesized directly from the QDD2.1_beta output, where only four primer pairs yielded PCR amplification but none of them produced consistently scorable products. These markers are currently being tested in copepod and goatfish samples from locations representative of their distribution range, for which separate reports are being prepared.

**Table 3:** Summary numbers for the post-sequencing selection of microsatellite loci from sequencing run 2.

Number	*H. longicornis*	*P. xiphias*	*O. cyanea*	*O. oliveri*
**Before Quality Control**				
Sequences	64916	75702	56233	59445
µsats	8101	9927	21795	20293
% with µsats	12.5	13.1	38.8	34.1
**After Quality Control**				
Low Stringency - consensus				
Sequences	62547	73140	54500	58088
µsats	103	168	790	734
µsats without RE^3^	73	109	429	729
Primer pairs	55	82	261	500
High Stringency - singletons				
Sequences	37753	45842	36174	40304
µsats	534	1027	4067	3887
µsats without RE^3^	372	633	1735	1661
Primer pairs	188	305	550	525
Total primer pairs	243	387	811	1025

3 RE – repetitive elements.

### Post-sequencing Selection of Microsatellite Loci via Bioinformatic Analyses in Fishes and Sea Stars

Following the same pipeline we identified microsatellite markers in nine additional libraries from eight additional species. We applied two strategies to select microsatellite loci. The first consisted of performing a low stringency QC and only designing primers from the contigs that had no similarity to repetitive elements. The second strategy was to use a very stringent QC (i.e. to select sequence regions with high base-call accuracy) and only designing primers in the singleton sequences that had no similarity to repetitive elements. The results are summarized in Tables 2 and 3. For the goatfish *M. vanicolensis* we identified 380 putative microsatellite loci. Similarly, we identified 875 and 850 candidate microsatellite markers from two hawkfish *P. arcatus* libraries (brown and pink morphotypes, respectively). For the sea star, *P. nodosus,* we identified 466 microsatellite loci and for the other sea star, *A. planci,* we identified 276 loci. Similarly, we identified 243 candidate microsatellite markers in the copepod *H. longicornis,* and 811 and 1025 candidate loci from the octopuses *O. cyanea* and *O. oliveri*, respectively.

## Discussion

Shallow genome pyrosequencing with as little as 1/32 of a PicoTiterPlate can deliver sufficient microsatellite loci for most ecological studies of non-model taxa. In the raw datasets, microsatellite-containing reads numbered in the thousands regardless of whether or not we enriched for microsatellites. Enrichment, however, did tend to produce a higher percentage of sequences with microsatellites (e.g., *Paracirrhites arcatus*, Tables 2 and 3). Here we show that applying simple bioinformatic selection tools prior to primer design will reduce laboratory time and costs relative to randomly testing subsets of potential primers.

The 454 technology is known to be highly variable in terms of the quality of the reads [26], [27]. Since the accuracy of base calling in the microsatellite flanking region impacts PCR amplification success, sequence quality is of primary importance. To the best of our knowledge, however, quality control such as we outline here has not been applied to previous 454 microsatellite studies. As with the most successful previous approaches prior to the advent of pyrosequencing (e.g. [15], [16], [28], [29]), projects using 454 sequencing should employ a rigorous initial quality control step. However, an obvious tradeoff exists because the chances of successful PCR amplification are increased with higher thresholds of sequence accuracy, but a very stringent quality control will drastically reduce the number of candidate microsatellite loci to test. Compared to methods based on mean sequence quality, the sliding window approach that we used (implemented in Clean_reads) results in a larger number of sequences in the clean dataset, because the 3′end of the sequences typically have the lowest quality, which reduces the overall mean quality score in otherwise robust sequences. An additional way to increase sequence accuracy is to design primers in the consensus sequences of the contigs where multiple sequence reads of the same locus can be used to compensate for base calling errors. Here, we designed primers from only the most accurate singletons and then relaxed the stringency of the QC to design primers from high-quality, consensus sequences of the contigs. Researchers might want to design primers in both of these pools or focus on just one. For instance, contigs may be the primary source of putative loci in enrichment preparation methods, whereas in shotgun projects, where a larger number of loci are sequenced at a lesser depth, a higher number of singletons is expected. Additionally, most errors on the 454 platform are associated with homopolymer-length calling [30]. Limiting the maximum homopolymer length allowed in the priming sites is a useful control to mitigate this issue. Our laboratory tests confirmed very high primer-to-marker conversion rates, which we attribute to the increase in template sequence accuracy.

A second, and less recognized, impediment to finding successful microsatellites primers is multicopy DNA regions such as transposable elements (transposons). These small repetitive DNA segments can insert themselves into new locations, and can account for a large portion of a genome. For example, transposable elements comprise up to 80% of some grass genomes and 66% of the human genomes, these values being most likely underestimations [31]–[33]. In fish, these elements have only been studied in a few model organisms for which genomic information is available, however all known families of transposons have been identified in this group and they seem to play an important role in genome evolution [34].

Genomic studies indicate that microsatellites are often found in close association with transposable elements [32], [35]–[37] which can lead to amplification of multicopy products rather than a single locus [8]. Repetitive elements have been studied in a variety of organisms and their sequences are available in public databases (e.g. Repbase) facilitating their identification in genomic sequencing datasets. Microsatellite projects on vertebrates, arthropods and many plant taxa can benefit from comparisons to the genomic information available from related genomes. To reduce the chances of targeting microsatellite loci associated with repetitive elements, we compared our sequences to databases of repetitive elements, which allowed us to remove sequences with REs, including retrotransposons and pseudogenes. The RE filter can be applied before or after microsatellite detection. Because taxonomic coverage of RE databases is limited, false negative discovery rates are likely to be high in most comparisons, as it is unlikely that all repetitive elements present in the genome of a focal taxon are represented in the RE databases. To apply a filter that does not depend on the availability of genomic resources, we also eliminated sequences that were partially homologous to other sequences in our datasets (i.e. sequences with BLAST hits for similarity with 80–95% identity). Alternative *de novo* detection methods of repetitive elements could be incorporated in the pipeline (e.g. *P-clouds* [33], [38]).

Logically, the probability of detecting a given locus is a function of its copy number in the genome, so highly repetitive elements have a disproportionate probability of being selected in random screening. That none of the primer pairs selected with our PSMS pipeline amplified multiple targets in the PCR tests (in contrast with the ones developed with previous methodologies) is an indication of the value of our approach.

We argue that researchers developing microsatellites markers from any massively parallel sequencing technology should take advantage of available bioinformatic tools and genomic resources to explore their sequence datasets. A small initial investment of computer time can provide extensive savings in terms of laboratory costs and time spent optimizing poorly performing primers and scoring markers that amplify repetitive regions.

## Supporting Information

Figure S1 Box plots depicting the sequence quality along the reads of *M.vanicolensis:* a) before quality control, b) after quality control with low stringency parameters (see methods), c) after quality control with high stringency parameters. The x-axis is the length of the sequencing reads expressed in bp and on the y-axis is the sequence quality as represented by Phred scores. In the boxplots, the black lines indicate median values, the dark red boxes below and above the black lines indicate the lower and upper quartiles respectively, and the light red boxes (a) and ends of the whiskers (b, c) represent the minimum and maximum quality scores at each position.
(TIF)
