# Supplementary figures and images for: Microsatellites for Next-Generation Ecologists: A Post-Sequencing Bioinformatics Pipeline
Source: PLoS One. 2013 Feb 12;8(2):e55990. doi: 10.1371/journal.pone.0055990 (PMC3570555; doi:10.1371/journal.pone.0055990)

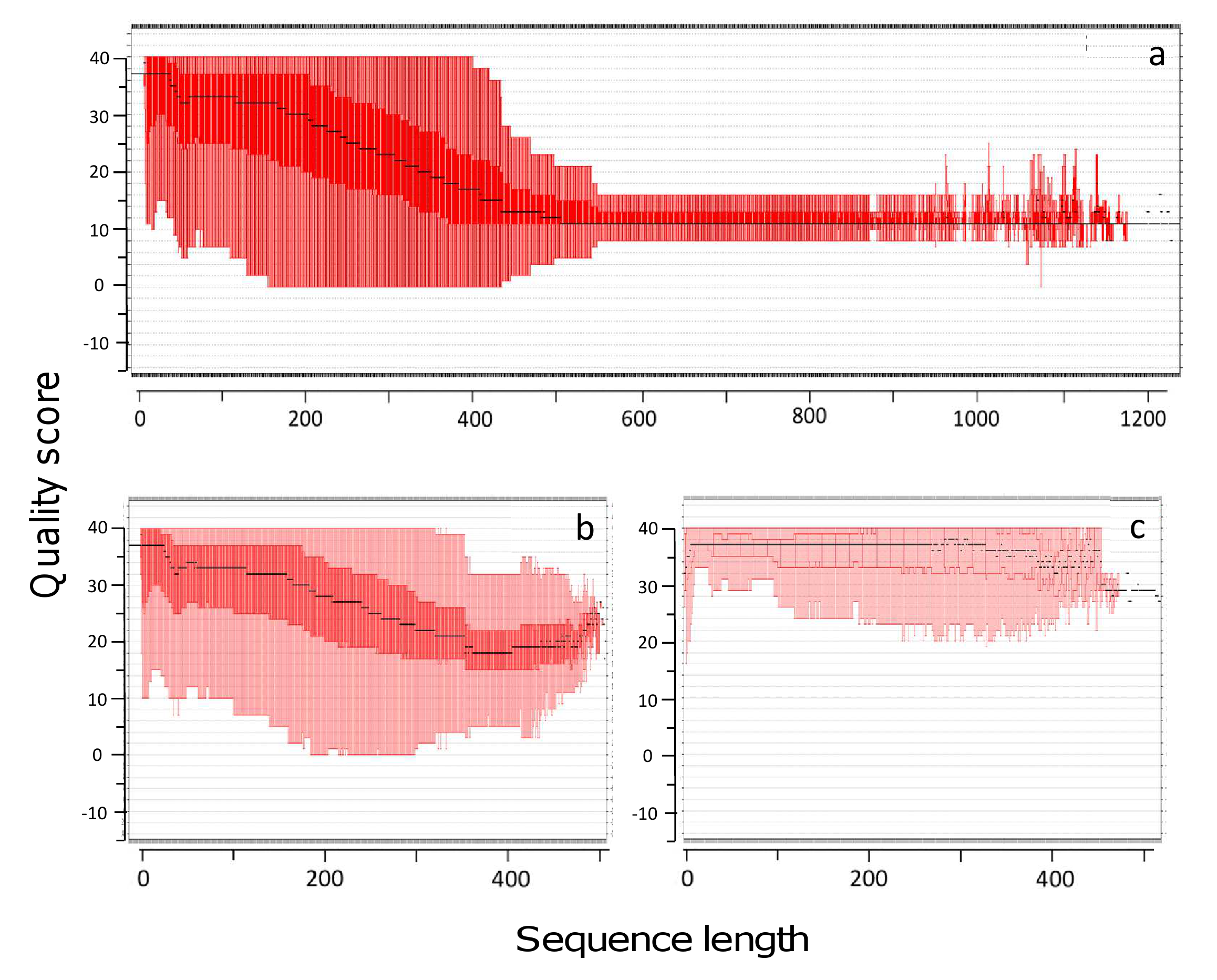

Supplement: Figure S1 — Box plots depicting the sequence quality along the reads of M.vanicolensis: a) before quality control, b) after quality control with low stringency parameters (see methods), c) after quality control with high stringency parameters. The x-axis is the length of the sequencing reads expressed in bp and on the y-axis is the sequence quality as represented by Phred scores. In the boxplots, the black lines indicate median values, the dark red boxes below and above the black lines indicate the lower and upper quartiles respectively, and the light red boxes (a) and ends of the whiskers (b, c) represent the minimum and maximum quality scores at each position. (TIF) [file pone.0055990.s001.tif]
